# Supplementary figures and images for: A pilot study: effect of irisin on trabecular bone in a streptozotocin-induced animal model of type 1 diabetic osteopathy utilizing a micro-CT
Source: PeerJ. 2023 Oct 17;11:e16278. doi: 10.7717/peerj.16278 (PMC10588705; doi:10.7717/peerj.16278)

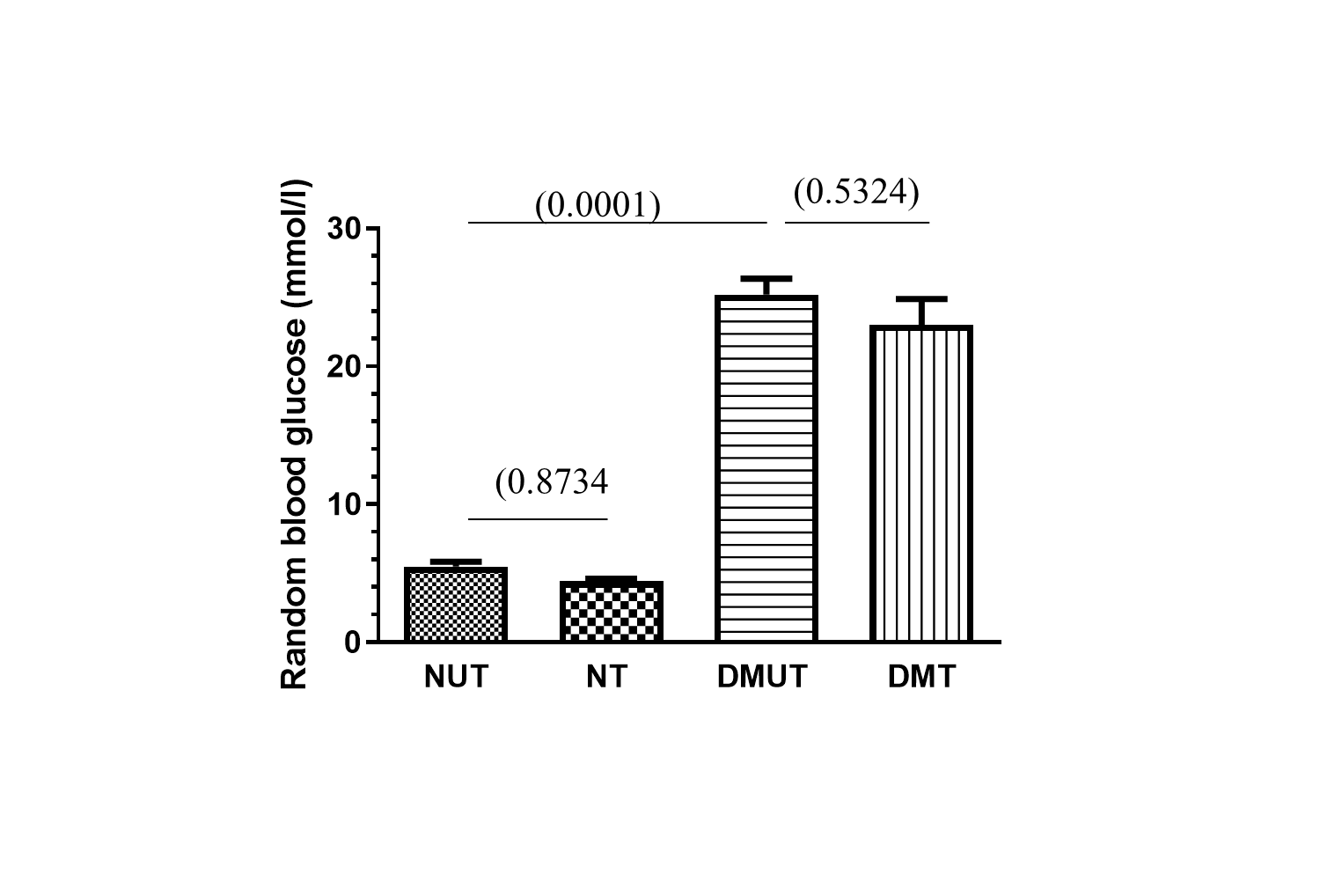

Supplement: Supplemental Information 2 [file peerj-11-16278-s002.png]
