# Supplementary material for: A pilot study: effect of irisin on trabecular bone in a streptozotocin-induced animal model of type 1 diabetic osteopathy utilizing a micro-CT
Source: PeerJ. 2023 Oct 17;11:e16278. doi: 10.7717/peerj.16278 (PMC10588705; doi:10.7717/peerj.16278)
Supplement: Supplemental Information 3 — Mean ± S.E between different groups related to trabecular bone parameters obtained using micro-CT. Gp. I—normal un-treated/NUT, Gp. II— normally treated (NT), Gp. III—diabetic un-treated (DMUT), and Gp. IV—diabetic treated (DMT). Trabecular separation Tb-Sp Gp (I–III = p < 0.05: Trabecular thickness Tb-Th Gp (II and III; II–IV = p < 0.05): Trabecular number Tb-N Gp (III and IV = p < 0.05): bone volume/total volume BV/TV Gp (I–III; II and III = p < 0.05): bone surface density BS/BV Gp (II and III; II–IV p < 0.05): Bone mineral density BMD Gp (I–III; II and III = p < 0.05). n = 3/Gp. [file peerj-11-16278-s003.pdf]

Groups	Lane	SOST	GAPDH	Normalized SOST expression
NUT	1	1924.175	1315.305	1.463
NUT	2	3355.974	1875.891	1.789
NUT	3	3064.853	1688.477	1.815
NT	4	4323.459	1514.184	2.855
NT	5	3252.325	1273.859	2.553
NT	6	3312.754	1166.251	2.841
DMUT	7	5940.711	2024.426	2.935
DMUT	8	7441.974	1831.841	4.063
DMUT	9	6037.69	1767.77	3.415
DMT	10	2746.79	2250.033	1.221
DMT	11	2424.104	2137.619	1.134
DMT	12	2025.121	1714.379	1.181

Suppl Data 1a: Densitometric measurements of SOST western blot

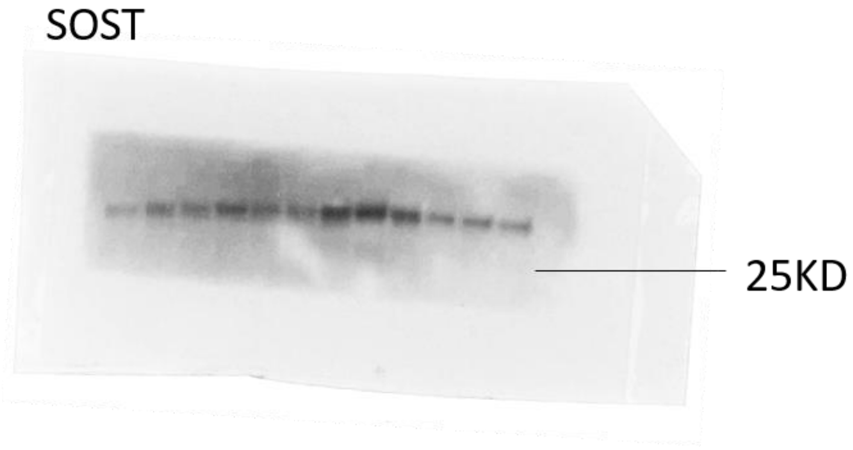

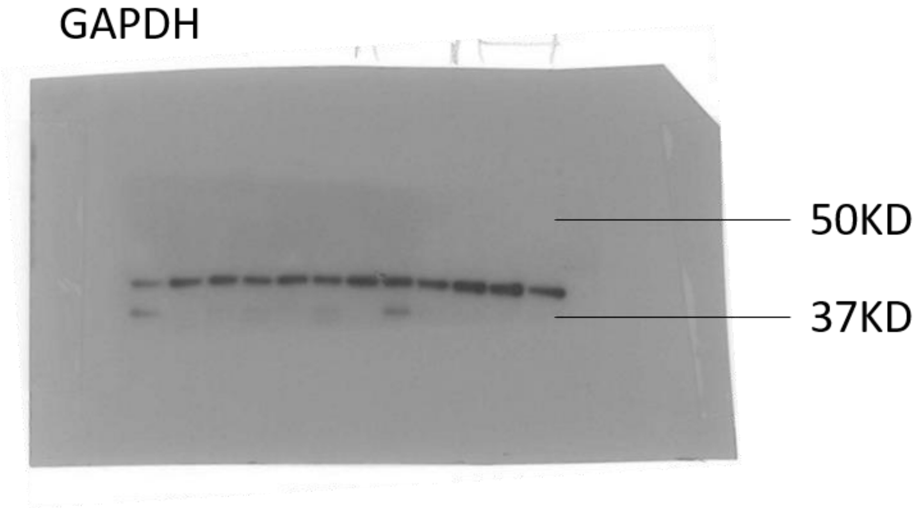

Supp Data 1b: Uncut Western blot for SOST & GAPDH
